# Bone Infarct as an Indicator of Acute Spinal Ischaemia

**DOI:** 10.1155/2020/9703625

**Published:** 2020-02-26

**Authors:** Laura López-Viñas, Kary Leonisa Quiñones-Coneo, Raquel Buenache-Espartosa, Juan Salvador Martínez-San-Millán, Gustavo Lorenzo-Sanz

**Affiliations:** ^1^Neurophysiology Department, La Princesa University Hospital, Madrid, Spain; ^2^Paediatric Neurology, Paediatrics Department, Ramon y Cajal Hospital, Alcala University, Madrid, Spain; ^3^Radiology Department, Ramon y Cajal Hospital, Madrid, Spain

## Abstract

Acute spinal cord infarct in childhood is extremely rare, generally secondary to spinal/cardiovascular surgery or severe vertebral injuries. However, spontaneous spinal cord infarct cases have been described. We present a clinical case of a teenager who developed an acute weakness and paraesthesia in lower limbs after playing piggyback. Laboratory tests and MRI (magnetic resonance imaging) were normal. During her hospital admission, her motor strength improved. After 10 days, MRI was repeated, and a bone infarct was observed. She was medicated with acetylsalicylic acid, and she completed a rehabilitation program.

## 1. Introduction

Acute myelopathy could be caused by several diseases, such as multiple sclerosis, infectious illness, ionizing radiation, rheumatological disorders, or spinal cord inflammation, although the cause is occasionally unknown [1].

Acute spinal cord infarct in childhood is extremely rare, generally secondary to spinal/cardiovascular surgery or severe vertebral injuries. However, spontaneous spinal cord infarct in childhood has been described. These are usually caused by procoagulant diseases, spinal vascular malformation, or minor injuries [2, 3].

If acute myelopathy is suspected, a compressive mechanical damage should be considered. In this case, a spinal cord MRI (magnetic resonance imaging) should be done, and it might be necessary to perform a brain MRI to rule out other diseases, such as acute disseminated encephalomyelitis, neuromyelitis optica, and multiple sclerosis (MS). Also, biochemical, immunological, and microbiological tests from CSF (cerebrospinal fluid) must be done to identify a possible infectious or autoimmune disease [3–5].

We present a clinical case based on a mild trauma in a teenager who developed an acute myelopathy secondary to spinal infarction.

## 2. Case Presentation

A teenage girl, without any medical history of interest, suffered an abrupt pain in the lumbar region, distal cold limbs, weakness, and paraesthesia in lower limbs, as well as walking difficulties. These symptoms appeared after playing piggyback.

At physical examination, she was unable to move her toes and to keep on her feet, but she could move her arms and the proximal area of her legs. After 4 hours, she suffered from bilateral motor loss in flexion and extension of her ankles (grade 1/5), and in her knees as well (grade 4/5), with preserved sensitivity, normal knee-jerk reflex, and hypoactive ankle-jerk reflex. Additionally, she had urine retention and overflow incontinence that was resolved spontaneously.

In an initial laboratory test, there were no data indicative of an infection or haemostatic abnormalities. MRI of the spinal cord and echo-Doppler of limbs were normal. Autoimmune disease test in CSF and blood coagulation study (G20210A mutation and methylenetetrahydrofolate reductase enzyme included) were normal as well. CSF cultures and serology were negative, with exception of M Immunoglobulin (Ig), which showed a positive result for *Mycoplasma pneumoniae*, without any seroconversion in G Ig.

**Figure 1:**
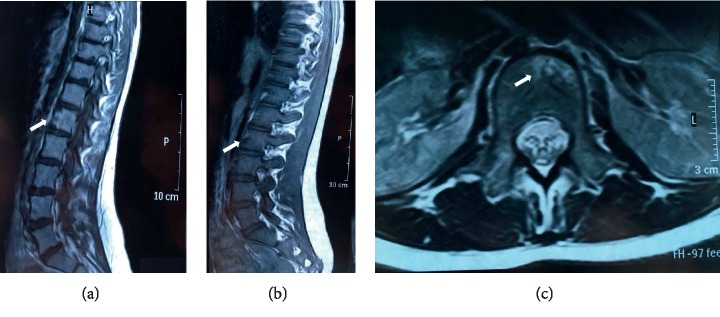
MRI of the dorsolumbar spinal cord. (a) Sagittal plane of the lumbar spinal cord, T1-weighted sequence with gadolinium contrast, and irregular increase of the signal on the front of vertebra L1 (arrow). (b) T2-weighted sequence, lack of recruitment in the same region (arrow). (c) Axial plane of vertebra L1, T1-weighted sequence with gadolinium contrast, and increase of the signal in the anterior third of the vertebral body (arrow) and conus medullaris.

During her hospital admission, her motor strength improved; however, she kept swaying walk. After 10 days, MRI was repeated, and an increase in bone density in conus medullaris and bone infarction in vertebral lumbar L1 (in T2-weighted sequence) were observed (Figure 1). She was medicated with acetylsalicylic acid, and she completed a rehabilitation program. In one-year follow-up, she improved her mobility, being able to walk by herself.

## 3. Discussion

Spinal cord infarction is a rare medical entity, but it can be the main cause of 14% of acute myelopathy [6]. The ischaemic mechanism could be elicited by an abrupt spinal movement over an underlying pathology. This often causes anterior or posterior spinal artery syndromes. In other cases, the main triggering factor is a prolonged hypotension with associated ischaemia, leading to a central cord or transverse infarction syndrome. The most likely cause is the spinal cord hypoperfusion, with greater risk for the lumbar region due to regional thickness of motor neurons and more structural mobility with secondary arterial compression [2, 3].

Spinal cord blood supply is an important key to highlight in the latter mechanism. Radicular arteries only irrigate anterior or posterior roots and dorsal ganglia, with little blood flow to the whole spinal cord. As with the presented clinical case, lower thoracic and lumbar regions of the spinal cord have poor vascular perfusion, depending exclusively on the artery of Adamkiewicz, being a particularly vulnerable area [7].

There are very few reports concerning spinal cord infarction in childhood. It has been associated to spinal cord or cardiovascular surgery [3], conditions that increase the risk of thrombophilia, such as prothrombin G20210A gene mutation, S protein deficit, or methylenetetrahydrofolate reductase mutation [8], myeloblastic leukemia [9], fibrocartilaginous thromboembolism secondary to trauma [10], or immunological disorders triggered by infectious process [3], among several triggering events.

Occasionally, just like in the aforementioned case, spinal cord ischaemia is associated with bone infarct of the vertebral body. This sign supports solid diagnostic criteria, highlighting the importance of neuroimaging study series.

In spinal cord MRI, we could observe “pencil-like” hyperintensity in the sagittal plane, in the T2-weighted sequence, and visualization of “H-” shape in the spinal cord in the axial plane, in the T1-weighted sequence with gadolinium contrast, which can be detected even more than 7 days from the injury. To detect these pathological findings, it is necessary to perform it 4 hours after the clinical onset. Radiological abnormalities are not specific of ischaemia, but sudden onset of symptomatology described above supports the diagnosis [11].

In the presence of an acute myelopathy associated with dorsolumbar pain in infancy, we must suspect that a spinal cord infarction could be taking place [1, 3]. It is very important to repeat MRI since it can show signs indicating a potential spinal cord injury and vertebral body damage. It allows us to establish a diagnosis and start an early treatment, decreasing neurological sequelae.
